# Mechanistic Insights of Gamma Sensory Stimulation: CSF Proteomic Analyses Reveal Changes in Myelin and Synaptic Proteins

**DOI:** 10.1002/alz.087390

**Published:** 2025-01-09

**Authors:** Monika Shpokayte, Kiran Pandey, Annabelle C Singer, Duc Doung, James J. Lah, Allan I. Levey, Nicholas T Seyfried, Zach Malchano, Ralph Kern, Mihaly Hajos

**Affiliations:** ^1^ Cognito Therapeutics, Cambridge, MA USA; ^2^ Emtherapro, Atlanta, GA USA; ^3^ Georgia Institute of Technology, Atlanta, GA USA; ^4^ Emory University, Atlanta, GA USA; ^5^ Emory University School of Medicine, Atlanta, GA USA

## Abstract

**Background:**

Preclinical investigations in Alzheimer’s disease (AD) have highlighted the efficacy of gamma sensory stimulation in mitigating AD‐related pathologies. Cognito Therapeutics, Inc. (Cambridge, MA) has designed the Sensory Stimulation System for safe at‐home usage, to induce EEG‐confirmed gamma oscillations as a potential treatment for AD. In the present study, unbiased proteomic analysis of patient derived CSF samples was conducted to characterize the underlying mechanism of action and assess treatment effects of gamma sensory stimulation in AD.

**Method:**

The FLICKER study (NCT03543878) recruited ten participants with MCI due to AD from the Emory Goizueta Alzheimer's Disease Research Center. The trial employed a delayed‐start study design with 4‐ or 8‐week daily gamma sensory stimulation. Cerebrospinal fluid (CSF) was collected at baseline, 4‐ and 8‐weeks and the proteome was analyzed (Emtherapro, Atlanta, GA) using tandem‐mass tag spectrometry. The CSF proteome of participants was mapped to previously established brain‐derived co‐expression modules (Johnson et al. 2022) of AD. Concordant and Discordant proteins were analyzed using Emtherpro’s internal AD proteome database.

**Result:**

A total of 2,785 CSF proteins were detected across all CSF samples. Differential expression analysis of proteins from baseline (N=5) vs. (N=5, 8 weeks) revealed 110 proteins that met the significance threshold. 60 proteins upregulated and 50 proteins downregulated as result of treatment. Treatment significantly impacted CSF proteins associated with AD‐related brain modules, including synapses, myelination, post‐synaptic density, and neurotransmitter regulation. Two key myelin‐related proteins that showed significant treatment time‐dependent changes over 8‐weeks of treatment were PLP1, a major structural component of myelin, and EV12A, a cell surface receptor primarily found on myelinating oligodendrocytes and precursor cells. Changes are also seen in cell surface receptors and molecules such as TENM3, EPHA7, and DSCAM which are involved in neuronal network failure, neurogenesis, and neuronal plasticity, respectively. Lastly, γSynuclein, a protein linked to neurofilament network integrity and memory dysfunction, exhibited significant decreases over 8 weeks.

**Conclusion:**

This study shows that sensory stimulation induces broad biomarker support, highlighting its impact on myelin changes, synaptic regulation, and network function. These findings contribute to our understanding of the underlying mechanisms of gamma sensory stimulation in the context of AD.

